# Trimethyl­phenyl­ammonium dibromidotriphenyl­stannate(IV)

**DOI:** 10.1107/S1600536809019722

**Published:** 2009-06-06

**Authors:** Quai Ling Yap, Kong Mun Lo, Seik Weng Ng

**Affiliations:** a Department of Chemistry, University of Malaya, 50603 Kuala Lumpur, Malaysia

## Abstract

The five-coordinate Sn atom in the title salt, [(CH_3_)_3_(C_6_H_6_)N][SnBr_2_(C_6_H_5_)_3_], exists in a distorted *trans*-C_3_SnBr_2_ trigonal-bipyramidal coordination geometry. In the crystal structure no obvious hydrogen bonding is observed.

## Related literature

The are few examples of dihalogenotriaryl­stannate salts having a counter-ion that does not participate in hydrogen bonding, which appears to assist in stabilizing the salt, see: Beckmann *et al.* (2002 ▶); Harrison *et al.* (1978 ▶); Kuhn *et al.* (2001 ▶); Ng (1995 ▶); Wharf & Simard (1991 ▶).
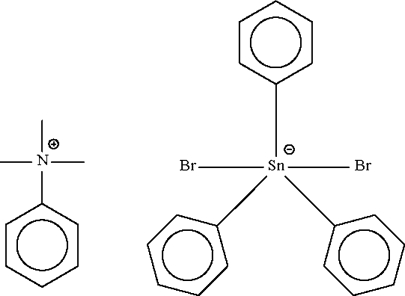


## Experimental

### 

#### Crystal data

(C_9_H_14_N)[SnBr_2_(C_6_H_5_)_3_]
*M* *_r_* = 646.02
Monoclinic, 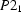

*a* = 9.0010 (1) Å
*b* = 16.7778 (2) Å
*c* = 9.2448 (1) Å
β = 111.003 (1)°
*V* = 1303.37 (3) Å^3^
*Z* = 2
Mo *K*α radiation
μ = 4.06 mm^−1^
*T* = 100 K
0.30 × 0.25 × 0.20 mm

#### Data collection

Bruker SMART APEX diffractometer
Absorption correction: multi-scan (*SADABS*; Sheldrick, 1996 ▶) *T*_min_ = 0.567, *T*_max_ = 0.746 (expected range = 0.337–0.444)
12525 measured reflections
5966 independent reflections
5889 reflections with *I* > 2σ(*I*)
*R*_int_ = 0.012

#### Refinement

*R*[*F* ^2^ > 2σ(*F* ^2^)] = 0.016
*wR*(*F* ^2^) = 0.042
*S* = 1.06
5966 reflections
280 parameters
1 restraint
H-atom parameters constrained
Δρ_max_ = 0.38 e Å^−3^
Δρ_min_ = −0.22 e Å^−3^
Absolute structure: Flack (1983 ▶), 2872 Friedel pairs
Flack parameter: 0.011 (3)

### 

Data collection: *APEX2* (Bruker, 2007 ▶); cell refinement: *SAINT* (Bruker, 2007 ▶); data reduction: *SAINT*; program(s) used to solve structure: *SHELXS97* (Sheldrick, 2008 ▶); program(s) used to refine structure: *SHELXL97* (Sheldrick, 2008 ▶); molecular graphics: *X-SEED* (Barbour, 2001 ▶); software used to prepare material for publication: *publCIF* (Westrip, 2009 ▶).

## Supplementary Material

Crystal structure: contains datablocks global, I. DOI: 10.1107/S1600536809019722/tk2456sup1.cif

Structure factors: contains datablocks I. DOI: 10.1107/S1600536809019722/tk2456Isup2.hkl

Additional supplementary materials:  crystallographic information; 3D view; checkCIF report

## Figures and Tables

**Fig. 1.**
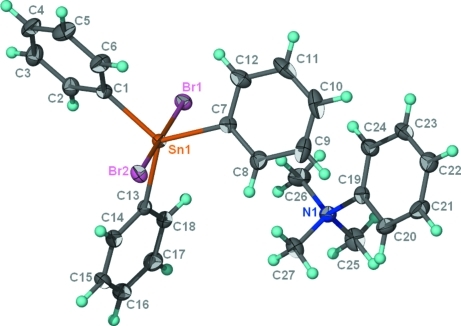
Thermal ellipsoid plot (Barbour, 2001) of [PhMe3N][SnBr2Ph3] at the 70% probability level. Hydrogen atoms are drawn as spheres of arbitrary radius.

## supplementary crystallographic information

### Experimental

Bis(4-dimethylaminopyridinium) dibromidotriphenylstannate (1.0 g, 1.6 mmol) and trimethylphenylammonium bromide (0.34 g, 1.6 mmol) were heated in ethanol for 1 hour.  Colorless crystals separated after a few days.

### Refinement

Hydrogen atoms were placed at calculated positions (C–H 0.95–0.98 Å) and were treated as riding on their parent atoms, with *U*(H) set to 1.2–1.5 times *U*_eq_(C).

### Crystal data

**Table:**

(C_9_H_14_N)[SnBr_2_(C_6_H_5_)_3_]	*F*(000) = 636
*M**_r_* = 646.02	*D*_x_ = 1.646 Mg m^−^^3^
Monoclinic, *P*2_1_	Mo *K*α radiation, λ = 0.71073 Å
Hall symbol: P 2yb	Cell parameters from 9930 reflections
*a* = 9.0010 (1) Å	θ = 2.4–28.3°
*b* = 16.7778 (2) Å	µ = 4.06 mm^−^^1^
*c* = 9.2448 (1) Å	*T* = 100 K
β = 111.003 (1)°	Block, colorless
*V* = 1303.37 (3) Å^3^	0.30 × 0.25 × 0.20 mm
*Z* = 2	

### Data collection

**Table:**

Bruker SMART APEX diffractometer	5966 independent reflections
Radiation source: fine-focus sealed tube	5889 reflections with *I* > 2σ(*I*)
graphite	*R*_int_ = 0.012
ω scans	θ_max_ = 27.5°, θ_min_ = 2.4°
Absorption correction: multi-scan (*SADABS*; Sheldrick, 1996)	*h* = −11→11
*T*_min_ = 0.567, *T*_max_ = 0.746	*k* = −21→21
12525 measured reflections	*l* = −12→12

### Refinement

**Table:**

Refinement on *F*^2^	Secondary atom site location: difference Fourier map
Least-squares matrix: full	Hydrogen site location: inferred from neighbouring sites
*R*[*F*^2^ > 2σ(*F*^2^)] = 0.016	H-atom parameters constrained
*wR*(*F*^2^) = 0.042	*w* = 1/[σ^2^(*F*_o_^2^) + (0.0283*P*)^2^ + 0.211*P*] where *P* = (*F*_o_^2^ + 2*F*_c_^2^)/3
*S* = 1.06	(Δ/σ)_max_ = 0.001
5966 reflections	Δρ_max_ = 0.38 e Å^−^^3^
280 parameters	Δρ_min_ = −0.22 e Å^−^^3^
1 restraint	Absolute structure: Flack (1983), 2872 Friedel pairs
Primary atom site location: structure-invariant direct methods	Flack parameter: 0.011 (3)

### Fractional atomic coordinates and isotropic or equivalent isotropic displacement parameters (Å^2^)

**Table:**

	*x*	*y*	*z*	*U*_iso_*/*U*_eq_	
Sn1	0.800735 (16)	0.499537 (6)	0.968485 (15)	0.01425 (4)	
Br1	0.50474 (2)	0.441885 (12)	0.78666 (2)	0.01891 (5)	
Br2	1.10719 (2)	0.544697 (12)	1.14791 (2)	0.01839 (5)	
N1	0.4280 (2)	0.36054 (11)	1.3067 (2)	0.0184 (3)	
C1	0.8550 (2)	0.52187 (12)	0.7653 (2)	0.0173 (4)	
C2	0.7539 (3)	0.56900 (13)	0.6462 (3)	0.0213 (4)	
H2	0.6596	0.5903	0.6549	0.026*	
C3	0.7901 (3)	0.58504 (15)	0.5146 (3)	0.0254 (5)	
H3	0.7209	0.6175	0.4346	0.030*	
C4	0.9258 (3)	0.55404 (16)	0.4999 (3)	0.0273 (5)	
H4	0.9495	0.5649	0.4097	0.033*	
C5	1.0273 (3)	0.50715 (18)	0.6166 (3)	0.0292 (5)	
H5	1.1205	0.4855	0.6060	0.035*	
C6	0.9933 (3)	0.49146 (15)	0.7499 (3)	0.0250 (5)	
H6	1.0645	0.4600	0.8304	0.030*	
C7	0.8524 (2)	0.38465 (13)	1.0804 (2)	0.0157 (4)	
C8	0.8729 (3)	0.37779 (14)	1.2360 (3)	0.0220 (4)	
H8	0.8638	0.4235	1.2931	0.026*	
C9	0.9068 (3)	0.30373 (16)	1.3081 (3)	0.0301 (5)	
H9	0.9167	0.2985	1.4135	0.036*	
C10	0.9261 (3)	0.23781 (15)	1.2269 (3)	0.0315 (6)	
H10	0.9494	0.1873	1.2764	0.038*	
C11	0.9113 (3)	0.24555 (14)	1.0730 (3)	0.0299 (5)	
H11	0.9264	0.2005	1.0176	0.036*	
C12	0.8743 (3)	0.31912 (14)	0.9998 (3)	0.0249 (5)	
H12	0.8641	0.3243	0.8944	0.030*	
C13	0.6992 (2)	0.59342 (13)	1.0628 (2)	0.0159 (4)	
C14	0.7844 (3)	0.66346 (13)	1.1201 (2)	0.0202 (4)	
H14	0.8905	0.6686	1.1227	0.024*	
C15	0.7154 (3)	0.72562 (13)	1.1732 (3)	0.0229 (4)	
H15	0.7747	0.7728	1.2120	0.027*	
C16	0.5604 (3)	0.71924 (14)	1.1700 (3)	0.0253 (5)	
H16	0.5126	0.7622	1.2041	0.030*	
C17	0.4761 (3)	0.64944 (15)	1.1163 (3)	0.0250 (5)	
H17	0.3710	0.6441	1.1161	0.030*	
C18	0.5447 (2)	0.58699 (13)	1.0626 (2)	0.0186 (4)	
H18	0.4855	0.5396	1.0256	0.022*	
C19	0.5292 (2)	0.28835 (13)	1.3666 (2)	0.0175 (4)	
C20	0.6100 (3)	0.28234 (14)	1.5255 (3)	0.0238 (5)	
H20	0.6051	0.3244	1.5924	0.029*	
C21	0.6975 (3)	0.21445 (15)	1.5849 (3)	0.0264 (5)	
H21	0.7536	0.2100	1.6933	0.032*	
C22	0.7040 (3)	0.15287 (14)	1.4874 (3)	0.0242 (4)	
H22	0.7628	0.1059	1.5290	0.029*	
C23	0.6246 (3)	0.15991 (13)	1.3294 (3)	0.0227 (4)	
H23	0.6302	0.1179	1.2626	0.027*	
C24	0.5367 (3)	0.22799 (13)	1.2673 (3)	0.0206 (4)	
H24	0.4826	0.2330	1.1586	0.025*	
C25	0.2875 (3)	0.35662 (15)	1.3594 (3)	0.0265 (5)	
H25A	0.3257	0.3534	1.4727	0.040*	
H25B	0.2234	0.3094	1.3147	0.040*	
H25C	0.2221	0.4046	1.3249	0.040*	
C26	0.3650 (3)	0.36554 (15)	1.1328 (3)	0.0317 (6)	
H26A	0.4542	0.3686	1.0958	0.048*	
H26B	0.2985	0.4132	1.0997	0.048*	
H26C	0.3012	0.3180	1.0894	0.048*	
C27	0.5184 (3)	0.43595 (14)	1.3676 (3)	0.0246 (4)	
H27A	0.6096	0.4394	1.3336	0.037*	
H27B	0.5563	0.4358	1.4810	0.037*	
H27C	0.4484	0.4819	1.3278	0.037*	

### Atomic displacement parameters (Å^2^)

**Table:**

	*U*^11^	*U*^22^	*U*^33^	*U*^12^	*U*^13^	*U*^23^
Sn1	0.01733 (6)	0.01231 (6)	0.01367 (6)	0.00070 (5)	0.00626 (4)	0.00004 (5)
Br1	0.01852 (9)	0.01789 (10)	0.01856 (10)	−0.00257 (7)	0.00452 (8)	−0.00274 (8)
Br2	0.01643 (9)	0.01616 (10)	0.01963 (10)	−0.00018 (7)	0.00289 (7)	−0.00003 (8)
N1	0.0194 (8)	0.0184 (9)	0.0166 (8)	0.0022 (7)	0.0056 (7)	0.0002 (7)
C1	0.0205 (9)	0.0167 (10)	0.0149 (9)	−0.0030 (7)	0.0064 (7)	−0.0023 (7)
C2	0.0207 (10)	0.0206 (10)	0.0219 (10)	−0.0020 (8)	0.0069 (8)	0.0007 (8)
C3	0.0246 (11)	0.0295 (12)	0.0183 (10)	−0.0072 (9)	0.0030 (8)	0.0035 (9)
C4	0.0313 (12)	0.0360 (13)	0.0164 (10)	−0.0127 (10)	0.0107 (9)	−0.0040 (9)
C5	0.0279 (11)	0.0385 (13)	0.0262 (11)	0.0014 (11)	0.0157 (9)	−0.0030 (11)
C6	0.0255 (10)	0.0293 (13)	0.0206 (10)	0.0051 (9)	0.0087 (8)	0.0017 (9)
C7	0.0108 (8)	0.0177 (9)	0.0160 (9)	0.0051 (7)	0.0014 (7)	0.0017 (8)
C8	0.0217 (10)	0.0222 (11)	0.0248 (11)	0.0014 (8)	0.0114 (9)	0.0022 (9)
C9	0.0276 (12)	0.0338 (13)	0.0323 (13)	0.0033 (10)	0.0148 (10)	0.0152 (11)
C10	0.0212 (10)	0.0212 (11)	0.0508 (16)	0.0022 (9)	0.0113 (11)	0.0155 (11)
C11	0.0252 (11)	0.0155 (10)	0.0466 (15)	0.0006 (9)	0.0101 (11)	−0.0062 (11)
C12	0.0246 (11)	0.0203 (11)	0.0264 (11)	−0.0016 (8)	0.0048 (9)	−0.0052 (9)
C13	0.0192 (9)	0.0152 (9)	0.0130 (8)	0.0030 (7)	0.0054 (7)	0.0030 (8)
C14	0.0229 (10)	0.0178 (10)	0.0197 (10)	−0.0006 (8)	0.0072 (8)	0.0007 (8)
C15	0.0329 (12)	0.0143 (10)	0.0213 (10)	−0.0016 (8)	0.0095 (9)	−0.0023 (8)
C16	0.0344 (12)	0.0202 (11)	0.0259 (11)	0.0079 (9)	0.0165 (10)	0.0015 (9)
C17	0.0263 (11)	0.0261 (11)	0.0270 (11)	0.0056 (9)	0.0150 (9)	0.0026 (9)
C18	0.0212 (9)	0.0176 (10)	0.0181 (9)	0.0003 (8)	0.0082 (8)	0.0013 (8)
C19	0.0155 (9)	0.0171 (10)	0.0198 (10)	0.0017 (7)	0.0061 (8)	0.0029 (8)
C20	0.0291 (11)	0.0254 (11)	0.0193 (11)	0.0052 (9)	0.0114 (9)	−0.0007 (9)
C21	0.0307 (12)	0.0310 (13)	0.0183 (10)	0.0081 (10)	0.0096 (9)	0.0039 (9)
C22	0.0240 (10)	0.0217 (11)	0.0273 (11)	0.0068 (8)	0.0099 (9)	0.0046 (9)
C23	0.0244 (10)	0.0167 (10)	0.0276 (11)	0.0001 (8)	0.0099 (9)	−0.0028 (9)
C24	0.0211 (10)	0.0184 (10)	0.0192 (10)	−0.0003 (8)	0.0034 (8)	−0.0011 (8)
C25	0.0165 (10)	0.0271 (12)	0.0373 (13)	0.0025 (9)	0.0115 (9)	0.0040 (10)
C26	0.0450 (14)	0.0278 (13)	0.0158 (10)	0.0150 (10)	0.0029 (10)	0.0027 (9)
C27	0.0257 (10)	0.0174 (10)	0.0304 (11)	−0.0012 (8)	0.0094 (9)	0.0006 (9)

### Geometric parameters (Å, °)

**Table:**

Sn1—Br1	2.7657 (2)	C13—C18	1.395 (3)
Sn1—Br2	2.7667 (2)	C13—C14	1.399 (3)
Sn1—C1	2.137 (2)	C14—C15	1.390 (3)
Sn1—C7	2.158 (2)	C14—H14	0.9500
Sn1—C13	2.156 (2)	C15—C16	1.389 (3)
N1—C27	1.500 (3)	C15—H15	0.9500
N1—C26	1.503 (3)	C16—C17	1.387 (4)
N1—C19	1.497 (3)	C16—H16	0.9500
N1—C25	1.511 (3)	C17—C18	1.394 (3)
C1—C2	1.396 (3)	C17—H17	0.9500
C1—C6	1.399 (3)	C18—H18	0.9500
C2—C3	1.394 (3)	C19—C24	1.385 (3)
C2—H2	0.9500	C19—C20	1.390 (3)
C3—C4	1.378 (4)	C20—C21	1.382 (3)
C3—H3	0.9500	C20—H20	0.9500
C4—C5	1.382 (4)	C21—C22	1.386 (3)
C4—H4	0.9500	C21—H21	0.9500
C5—C6	1.397 (3)	C22—C23	1.382 (3)
C5—H5	0.9500	C22—H22	0.9500
C6—H6	0.9500	C23—C24	1.391 (3)
C7—C12	1.381 (3)	C23—H23	0.9500
C7—C8	1.388 (3)	C24—H24	0.9500
C8—C9	1.391 (3)	C25—H25A	0.9800
C8—H8	0.9500	C25—H25B	0.9800
C9—C10	1.382 (4)	C25—H25C	0.9800
C9—H9	0.9500	C26—H26A	0.9800
C10—C11	1.387 (4)	C26—H26B	0.9800
C10—H10	0.9500	C26—H26C	0.9800
C11—C12	1.389 (3)	C27—H27A	0.9800
C11—H11	0.9500	C27—H27B	0.9800
C12—H12	0.9500	C27—H27C	0.9800
			
C1—Sn1—C7	120.02 (8)	C18—C13—Sn1	120.68 (16)
C1—Sn1—C13	119.42 (8)	C14—C13—Sn1	120.82 (14)
C7—Sn1—C13	120.54 (7)	C15—C14—C13	120.7 (2)
C1—Sn1—Br2	89.20 (5)	C15—C14—H14	119.7
C13—Sn1—Br2	91.91 (5)	C13—C14—H14	119.7
C7—Sn1—Br2	87.71 (5)	C16—C15—C14	120.5 (2)
C1—Sn1—Br1	90.06 (5)	C16—C15—H15	119.8
C13—Sn1—Br1	92.62 (5)	C14—C15—H15	119.8
C7—Sn1—Br1	88.51 (5)	C15—C16—C17	119.3 (2)
Br1—Sn1—Br2	175.151 (7)	C15—C16—H16	120.4
C27—N1—C26	107.47 (18)	C17—C16—H16	120.4
C27—N1—C19	111.54 (16)	C16—C17—C18	120.5 (2)
C26—N1—C19	112.77 (17)	C16—C17—H17	119.8
C27—N1—C25	108.64 (17)	C18—C17—H17	119.8
C26—N1—C25	107.97 (18)	C17—C18—C13	120.6 (2)
C19—N1—C25	108.32 (17)	C17—C18—H18	119.7
C2—C1—C6	118.6 (2)	C13—C18—H18	119.7
C2—C1—Sn1	120.17 (15)	C24—C19—C20	121.1 (2)
C6—C1—Sn1	121.19 (15)	C24—C19—N1	120.93 (18)
C3—C2—C1	120.5 (2)	C20—C19—N1	117.91 (19)
C3—C2—H2	119.8	C19—C20—C21	119.2 (2)
C1—C2—H2	119.8	C19—C20—H20	120.4
C4—C3—C2	120.4 (2)	C21—C20—H20	120.4
C4—C3—H3	119.8	C22—C21—C20	120.4 (2)
C2—C3—H3	119.8	C22—C21—H21	119.8
C5—C4—C3	120.0 (2)	C20—C21—H21	119.8
C5—C4—H4	120.0	C21—C22—C23	119.8 (2)
C3—C4—H4	120.0	C21—C22—H22	120.1
C4—C5—C6	120.2 (2)	C23—C22—H22	120.1
C4—C5—H5	119.9	C22—C23—C24	120.6 (2)
C6—C5—H5	119.9	C22—C23—H23	119.7
C5—C6—C1	120.4 (2)	C24—C23—H23	119.7
C5—C6—H6	119.8	C19—C24—C23	118.8 (2)
C1—C6—H6	119.8	C19—C24—H24	120.6
C12—C7—C8	120.1 (2)	C23—C24—H24	120.6
C12—C7—Sn1	120.01 (15)	N1—C25—H25A	109.5
C8—C7—Sn1	119.79 (15)	N1—C25—H25B	109.5
C9—C8—C7	119.7 (2)	H25A—C25—H25B	109.5
C9—C8—H8	120.2	N1—C25—H25C	109.5
C7—C8—H8	120.2	H25A—C25—H25C	109.5
C10—C9—C8	120.2 (2)	H25B—C25—H25C	109.5
C10—C9—H9	119.9	N1—C26—H26A	109.5
C8—C9—H9	119.9	N1—C26—H26B	109.5
C9—C10—C11	119.8 (2)	H26A—C26—H26B	109.5
C9—C10—H10	120.1	N1—C26—H26C	109.5
C11—C10—H10	120.1	H26A—C26—H26C	109.5
C12—C11—C10	120.1 (2)	H26B—C26—H26C	109.5
C12—C11—H11	120.0	N1—C27—H27A	109.5
C10—C11—H11	120.0	N1—C27—H27B	109.5
C7—C12—C11	120.0 (2)	H27A—C27—H27B	109.5
C7—C12—H12	120.0	N1—C27—H27C	109.5
C11—C12—H12	120.0	H27A—C27—H27C	109.5
C18—C13—C14	118.46 (19)	H27B—C27—H27C	109.5
			
C13—Sn1—C1—C2	−38.04 (19)	C10—C11—C12—C7	0.1 (3)
C7—Sn1—C1—C2	143.32 (15)	C1—Sn1—C13—C18	113.96 (16)
Br2—Sn1—C1—C2	−129.79 (16)	C7—Sn1—C13—C18	−67.41 (18)
Br1—Sn1—C1—C2	55.01 (16)	Br2—Sn1—C13—C18	−155.88 (16)
C13—Sn1—C1—C6	140.49 (17)	Br1—Sn1—C13—C18	22.41 (16)
C7—Sn1—C1—C6	−38.1 (2)	C1—Sn1—C13—C14	−63.53 (18)
Br2—Sn1—C1—C6	48.75 (17)	C7—Sn1—C13—C14	115.10 (17)
Br1—Sn1—C1—C6	−126.46 (17)	Br2—Sn1—C13—C14	26.62 (16)
C6—C1—C2—C3	0.2 (3)	Br1—Sn1—C13—C14	−155.09 (16)
Sn1—C1—C2—C3	178.79 (16)	C18—C13—C14—C15	−1.0 (3)
C1—C2—C3—C4	0.5 (3)	Sn1—C13—C14—C15	176.54 (16)
C2—C3—C4—C5	−0.4 (4)	C13—C14—C15—C16	−0.2 (3)
C3—C4—C5—C6	−0.4 (4)	C14—C15—C16—C17	1.4 (3)
C4—C5—C6—C1	1.1 (4)	C15—C16—C17—C18	−1.5 (3)
C2—C1—C6—C5	−1.0 (3)	C16—C17—C18—C13	0.4 (3)
Sn1—C1—C6—C5	−179.56 (19)	C14—C13—C18—C17	0.9 (3)
C1—Sn1—C7—C12	−19.52 (19)	Sn1—C13—C18—C17	−176.66 (16)
C13—Sn1—C7—C12	161.86 (15)	C27—N1—C19—C24	−131.5 (2)
Br2—Sn1—C7—C12	−107.26 (16)	C26—N1—C19—C24	−10.4 (3)
Br1—Sn1—C7—C12	69.69 (16)	C25—N1—C19—C24	109.0 (2)
C1—Sn1—C7—C8	156.62 (15)	C27—N1—C19—C20	51.2 (2)
C13—Sn1—C7—C8	−22.00 (19)	C26—N1—C19—C20	172.2 (2)
Br2—Sn1—C7—C8	68.87 (15)	C25—N1—C19—C20	−68.3 (2)
Br1—Sn1—C7—C8	−114.17 (15)	C24—C19—C20—C21	−0.8 (3)
C12—C7—C8—C9	−3.6 (3)	N1—C19—C20—C21	176.6 (2)
Sn1—C7—C8—C9	−179.74 (16)	C19—C20—C21—C22	−0.4 (4)
C7—C8—C9—C10	2.5 (3)	C20—C21—C22—C23	1.1 (4)
C8—C9—C10—C11	−0.1 (3)	C21—C22—C23—C24	−0.7 (4)
C9—C10—C11—C12	−1.2 (4)	C20—C19—C24—C23	1.1 (3)
C8—C7—C12—C11	2.3 (3)	N1—C19—C24—C23	−176.13 (19)
Sn1—C7—C12—C11	178.46 (17)	C22—C23—C24—C19	−0.4 (3)
